# Potential promising anticancer applications of β-glucans: a review

**DOI:** 10.1042/BSR20231686

**Published:** 2024-01-09

**Authors:** Seyed Mostafa Noorbakhsh Varnosfaderani, Farnoosh Ebrahimzadeh, Mahsa Akbari Oryani, Saeed Khalili, Faezeh Almasi, Reza Mosaddeghi Heris, Zahra Payandeh, Chen Li, Mohsen Nabi Afjadi, Armina Alagheband Bahrami

**Affiliations:** 1 Department of Radiology, Afzalipour Hospital, Kerman University of Medical Science, Kerman, Iran; 2 Department of Internal Medicine, Faculty of Medicine, Mashhad University of Medical Sciences, Mashhad, Iran; 3 Department of Pathology, Faculty of Medicine, Mashhad University of Medical Sciences, Mashhad, Iran; 4 Department of Biology Sciences, Shahid Rajaee Teacher Training University, Tehran, Iran; 5 Pharmaceutical Biotechnology Lab, Department of Microbial Biotechnology, School of Biology and Center of Excellence in Phylogeny of Living Organisms, College of Science, University of Tehran, Tehran, Iran; 6 Neurosciences Research Center, Tabriz University of Medical Sciences, Tabriz, Iran; 7 Immunology Research Center, Tabriz University of Medical Sciences, Tabriz, Iran; 8 Department of Biology, Chemistry, Pharmacy, Free University of Berlin, Berlin, Germany; 9 Department of Biochemistry, Faculty of Biological Sciences, Tarbiat Modares University, Tehran, Iran; 10 Department of Biotechnology, School of Advanced Technologies in Medicine, Shahid Beheshti University of Medical Sciences, Tehran

**Keywords:** β-glucans, antitumor, bioactive polysaccharides, cancer

## Abstract

β-Glucans are valuable functional polysaccharides distributed in nature, especially in the cell walls of fungi, yeasts, bacteria, and cereals. The unique features of β-glucans, such as water solubility, viscosity, molecular weight, and so on, have rendered them to be broadly applied in various food systems as well as in medicine to improve human health. Moreover, inhibition of cancer development could be achieved by an increase in immune system activity via β-glucans. β-glucans, which are part of a class of naturally occurring substances known as biological response modifiers (BRMs), have also shown evidence of being anti-tumorogenic, anti-cytotoxic, and anti-mutagenic. These properties make them attractive candidates for use as pharmaceutical health promoters. Along these lines, they could activate particular proteins or receptors, like lactosylceramide (LacCer), Dickin-1, complement receptor 3 (CR3), scavenge receptors (SR), and the toll-like receptor (TLR). This would cause the release of cytokines, which would then activate other antitumor immune cells, like macrophages stimulating neutrophils and monocytes. These cells are biased toward pro-inflammatory cytokine synthesis and phagocytosis enhancing the elicited immunological responses. So, to consider the importance of β-glucans, the present review introduces the structure characteristics, biological activity, and antitumor functions of fungal β-glucans, as well as their application.

## Background

Polysaccharides are made to a large extent by all living organisms. These compounds are highly diverse in terms of chemical structure, physiological function, and their applicability in various industries such as food engineering, plastics, and cosmetics. Therefore, polysaccharides are renewable resources with significant potential for use by humans and can be found as homopolysaccharides and heteropolysaccharides. Many of these compounds are D-glucose monomers linked linearly or branched through α- or β-glycosidic bonds. In addition to the basic structure, the side chains comprise varied substituent groups, such as acyl groups, amino acids, and inorganic compounds [1]. One of these polysaccharide compounds produced by eukaryotes and prokaryotes is β-glucans. β-Glucans are known as functional and bioactive food ingredients through their biological activities, including hypoglycemic, immunomodulatory, hypocholesterolemic, antioxidant, and anti-inflammatory activities [2].

Owing to the excellent rheological attributes associated with the high viscosity of β-glucan at low concentrations, this polysaccharide can serve as a valuable food compound in products such as soups, sauces, and beverages to improve the sensory and taste characteristics of the products. On the other hand, due to their special physicochemical characteristics, β-D-glucan polysaccharides have a wide application in various industries such as agriculture and plant breeding, cosmetics, and medicine [3]. As mentioned above, they are one of the most valuable fungal compounds making them widely used today in medicine. Researchers believe that understanding the real value of β-glucans and their potential contribution to life improvement can help enhance health. So, β-glucans are employed in many research studies due to their biological activities, as well as their pharmacological and therapeutic properties.

The most important characteristics that determine the biological function of β-glucans are size, initial structure, amount of branching, molecular weight, degree of polymerization, purity, and more complex features such as bonding [4,5]. In this line, β-glucans can structurally be linear, annular, or branched with different bioactivity. These polysaccharides, which are comprised of D-glucose monomers, are connected by β-glycosidic bonds and are abundant in yeasts, fungi (such as mushrooms), several bacteria, seaweeds, and cereals (oats and barley).

**Figure 1:**
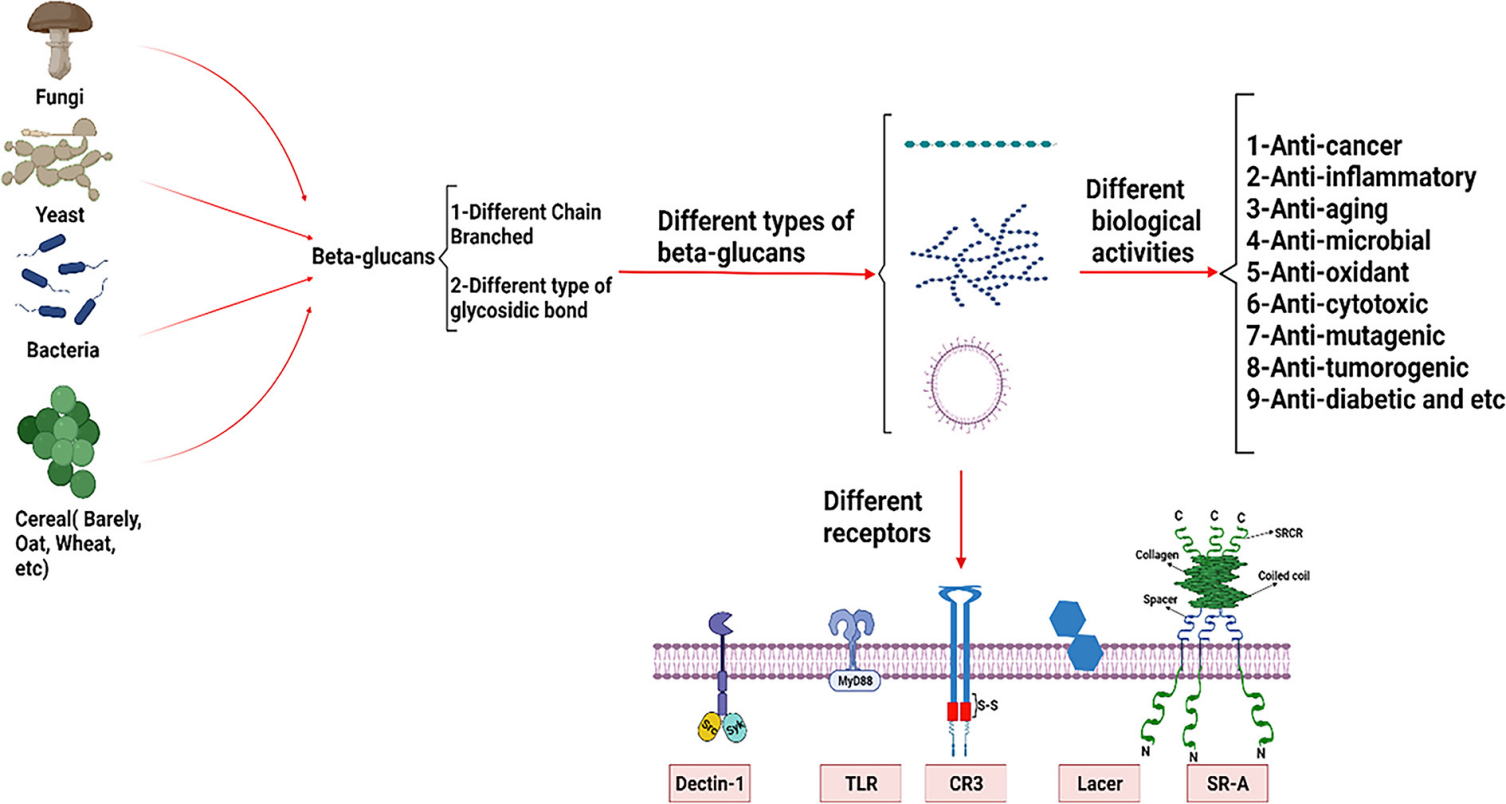
The structure and anticancer activity of β-glucans Different β-glucans show various biological activities and antitumor functions. They could trigger the secretion of cytokines through activating/inactivating specific receptors, such as Dickin-1, the toll-like receptor (TLR), complement receptor 3 (CR3), scavenge receptors (SR), and lactosylceramide (LacCer). Finally, improving the immune responses.

β-Glucans, belonging to a group of naturally occurring compounds called biological response modifiers (BRMs) have been also demonstrated to be anti-cytotoxic, anti-mutagenic, and anti-tumorogenic, which makes them promising candidates for pharmacological promoters of health. In this line, they could activate specific receptors or proteins, such as Dickin-1, the toll-like receptor (TLR), complement receptor 3 (CR3), scavenge receptors (SR), and lactosylceramide (LacCer), triggering the secretion of cytokines and subsequently activating other antitumor immune cells such as macrophages stimulating neutrophils and monocytes. These cells favor phagocytosis and the production of pro-inflammatory cytokines. Thus, they could improve the triggered immune responses [6]. According to the mentioned β-glucans’ interesting importance in human health, the present review discusses the structure characteristics, biological activity, and antitumor functions of fungal β-glucans, as well as their application (Figure 1).

## General characterization of β-glucans and functions thereof

**Figure 2:**
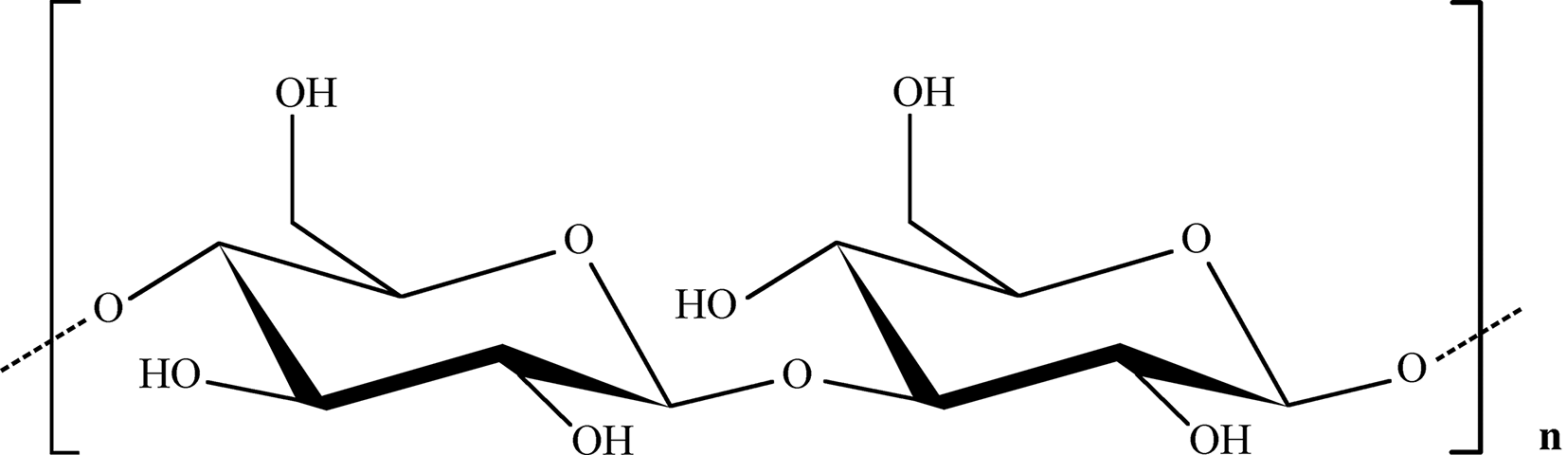
Linear β-(1,3)-D-glucan This compound has a long chain of D-glycopyranosyl units in its backbone, which are connected by 1→3 linkages, and has a side chain of β-glucopyranosyl with 1→6 linkages, which is randomly attached to the main structure.

β-Glucan is a water-soluble, non-starch polysaccharide existing in the cell wall of cereals such as barley, oats, wheat, rye, sorghum, rice, yeasts, bacteria, and algae. Moreover, there are hundreds of structurally and functionally different glucans, which are rooted in various natural sources, including yeast, mushrooms, bacteria, seaweed, and grain. Each type of β-glucan consists of a diverse molecular backbone, branching level, and molecular weight, affecting its solubility and physiological impact. Generally, β-glucans consist of glucose molecules linked together by a (1-3), (1,4), or (1,6) linear β-glycosidic chain, although different extraction techniques can result in their different conformations and subsequently, different biological properties. Although a wide range of β-D-glucans (with rich structural diversity) needs precise characterization, usually, fungal β-glucans have a (1-3) linked backbone with (1-6) side branches, while bacterial β-glucans tend to have (1-4) side branches [7]. Overall, the chemical structures of β-glucans can be classified into some important general groups generally divided into linear, branched, and cyclic glucans as in Figure 2.

This form of β-glucans can be found in prokaryotes and eukaryotes and can be extracted from grains, fungi, algae, and yeasts. This compound has a long chain of D-glycopyranosyl units in its backbone, which are connected by 1→3 linkages, and has a side chain of β-glucopyranosyl with 1→6 linkages, which is randomly attached to the main structure [7]. Although oat and wheat β-glucans are abundant in the cell walls of grains, they are structurally different from the β-glucans in yeasts and fungi. The chemical structure of oats-isolated β-glucans represents a linear structure formed by glucose units, which are connected by β (1-3) and β (1-4) bonds, and have short branches. The β (1-4) bonds correspond to 70% of the glycosidic bonds and appear in the sequence of two or three glucose units interrupted by a β (1-3) [8].

Many bacteria, for example, human, plant, and animal pathogens, produce extracellular polysaccharides, named exopolysaccharides as linear (1→3)-β-glucans, that production of some requires the coexistence of bacteria and plants. In addition, exopolysaccharides can be used as a gel and an emulsifier. *Rhizobium* and *Agrobacterium* are bacteria that can produce exopolysacharides under physiological conditions. An example of the mentioned compounds is Curdlan, which is produced by Agrobacterium species [9]. Curdlan is a high-molecular-weight polysaccharide comprising of β-(1→3)-linked glucose units and has the exceptional feature of forming an elastic gel upon heating its aqueous suspension. This polysaccharide, as a gel-forming agent, has broad applications in various industries, particularly the food, construction, and pharmaceutical industries [10]. For instance, Higashi et al. have studied the anti-tumor effect of this linear (1→3)-β-glucan. It has been reported that curdlan could induce DC-mediated Th17 differentiation and upregulate Jagged1 mRNA expression in THP-1 and human monoclonal-dendritic cells (Mo-DCs). In this regard, through *Jagged1* activation in human dendritic cells, curdlan polarizes the human DC-mediated Th17 suppressing tumor activity [11]. Other linear (1→3)-β-glucans include Pachymaran/Pachyman, Pustulan β-glucans (from *Saccharomyces cerevisiae*), Phycarine, and two Seaweed/Algal β-Glucans named Paramylon and Phycarine [12,13].

## Linear β-(1,3;1,4)-D-glucans

**Figure 3:**
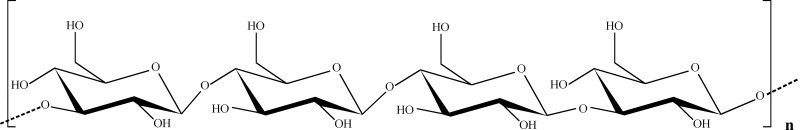
Linear β-(1,3;1,4)-D-glucan This type of glucan is mainly found in rice, wheat, cereal grains, and oats, serving as critical dietary fibers.

As shown in Figure 3, These glucans are mainly found in cereal grains (such as oats and barley), and marine algae (such as brown algae). Cereal β-Glucans include the Barley β-glucan [14], Oat β-glucan, and Wheat β-glucan [15]. Lichenan/Lichenin is one of the linear (1,3;1,4) β-glucan derived from *Cetraria islandica as a* marine alga [16].

The chemo-protective and anti-mutagenic activity of Barley β-glucan on CHO-K1 and HTC cell lines decreased the cell viability of different cancer cells [17]. Moreover, the low molecular weight (MW) of oat β-glucan has been reported to have anti-tumor activity [18].

## Side-chain-branched β-(1,3;1,6)-D-glucans

**Figure 4:**
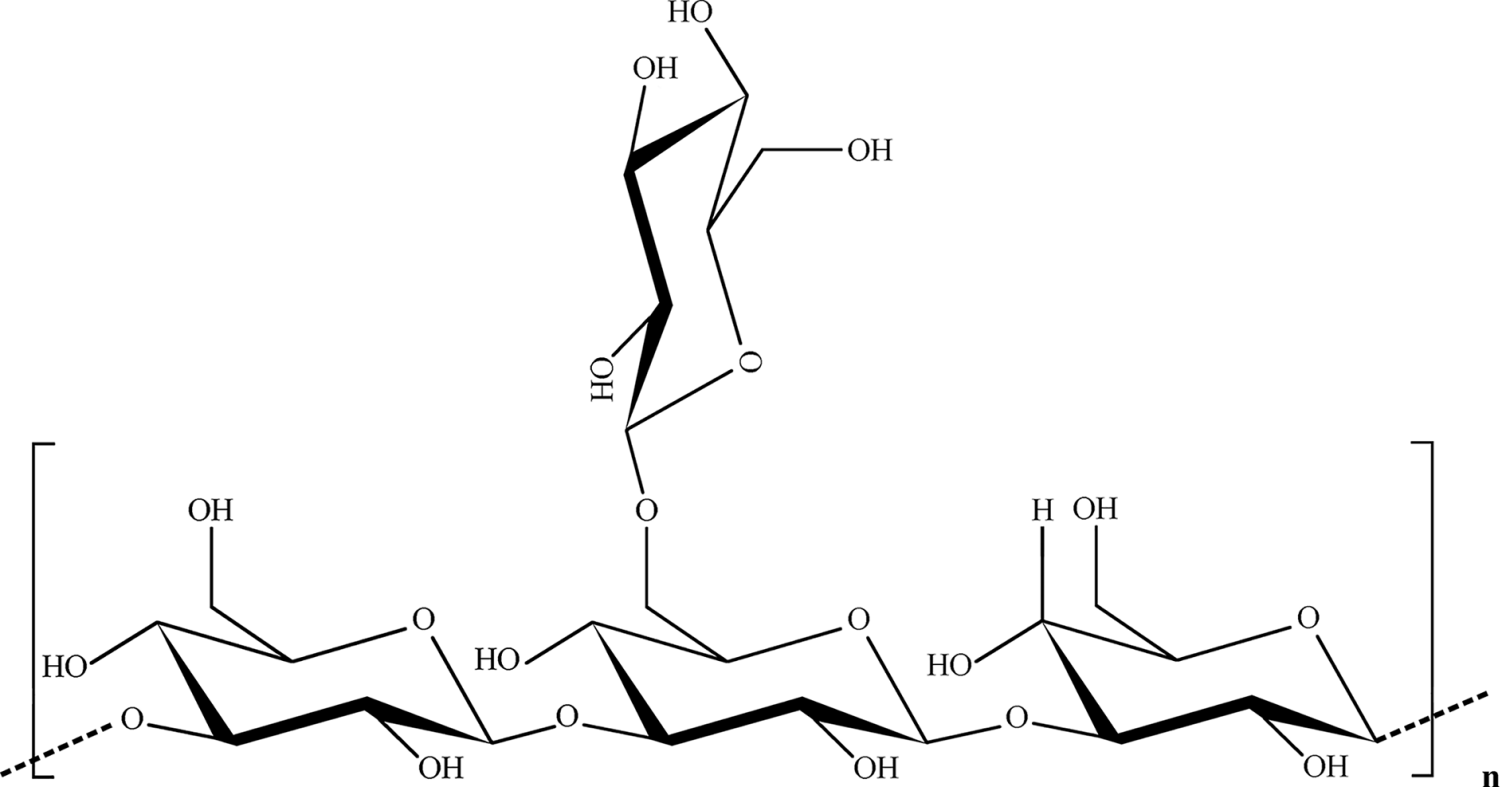
The side chain branched β-(1,3;1,6)-D-glucan Some fungal β-glucans and various yeast β-glucans have this type of glucan.

As shown in Figure 4, some fungal β-Glucans **(**T-4-N, pleura, tylopilan, etc.), and various yeast β-Glucans (zymosan, metafiction, yestimun, and Yerevan) have branched β-(1,3;1,6) structures. Various *in vitro* and *in vivo* studies have reported that yeast-derived (1,3;1,6) β-glucans could lead to antitumor activity via activation of macrophages and NK cells [19] and delay tumor progression [20] without toxicity in normal mouse cells. For instance, the β-(1,3;1,6)-D-Glucans, produced by *Diaporthe* sp. *Endophytes*, are shown to have antiproliferative activity against human breast carcinoma and hepatocellular carcinoma cells [21].

## Side-chain-branched β-(1,4;1,6)-D-glucans

**Figure 5:**
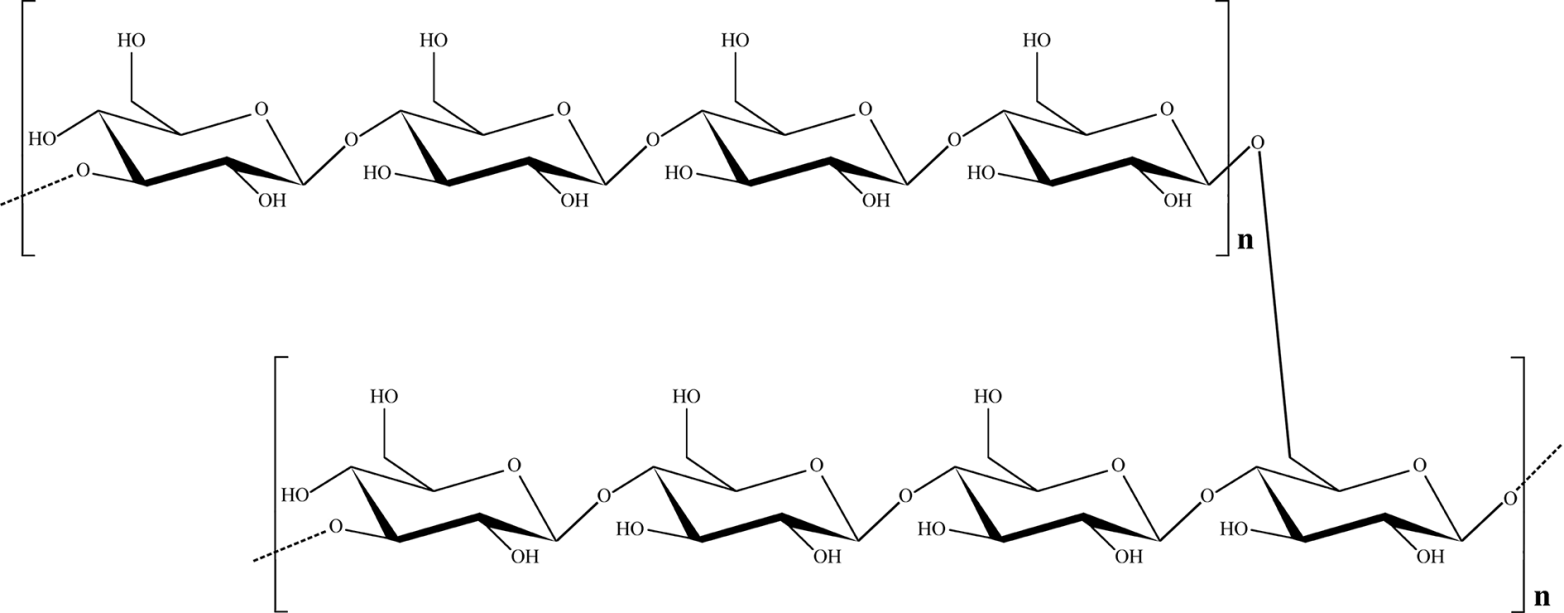
Side-chain-branched β-(1,4;1,6)-D-glucan Some yeast and fungal β-glucans contain side branches.

Glucans from *Xanthoria parietina* have been reported to have branched β-(1,4;1,6) structures (Figure 5) and macrophage-stimulating activity via activation of the dectin-2 receptor [22]. In addition, Su et al. have investigated the structural features and anti-inflammatory activity of water-soluble polysaccharides isolated from *Grifola frondosa* (GF). Accordingly, evaluation of receptor involvement has demonstrated that branched (1,4;1,6)-glucan in GF may contribute to its anti-inflammatory activity through interaction with TLR2 receptors [23].

## Cyclic β-(1,3;1,6)-glucans

**Figure 6:**
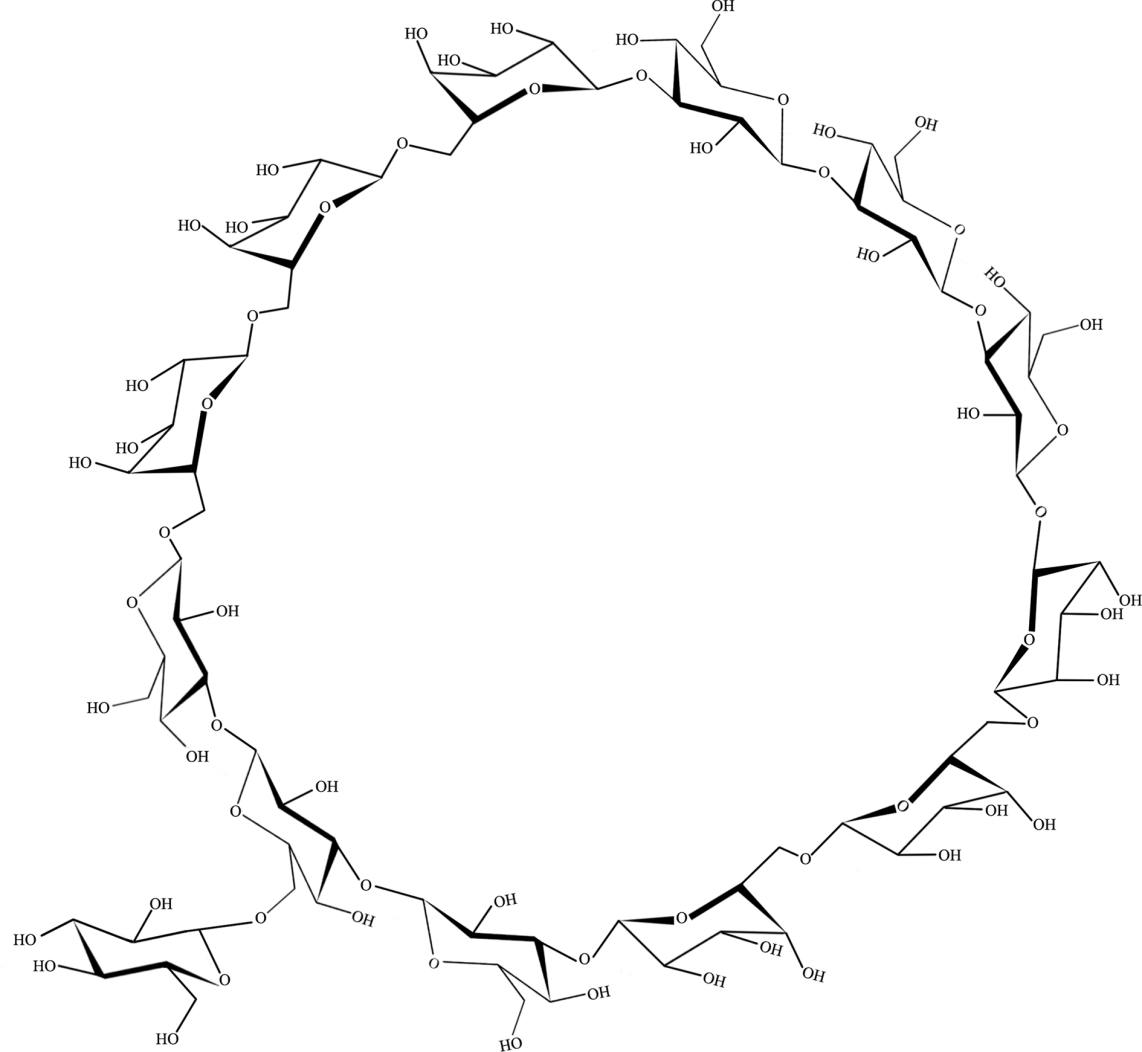
Cyclic β-(1,3;1,6)-glucan These glucans are cell-associated polysaccharides that are uniquely expressed in bacteria of the Rhizobiaceae family.

Cyclic β-glucans (Figure 6) are cell-associated polysaccharides that are uniquely expressed in bacteria of the Rhizobiaceae family (mostly *Bradyrhizobium japonicum* genus). These polysaccharides are cell surface carbohydrates, which are considered major cell envelope constituents. The cyclic β-(1,3;1,6)-glucans are promising encapsulating agents that could be used as drug delivery carriers for antitumor drugs such as betulinic acid (BA) [24].

## Structure–activity relationships of β-glucans in antitumor activity

The peculiarity of the actions of each type of β-glucan varies with its conformation, molecular mass, solubility, and the degree of branch positioning. The β-glucans with lower branching and lower polymerization (such as β-glucans from plants with β-(1-4) binding) are characterized as soluble, whereas insoluble ones such as β-glucans isolated from fungi with type bonds β-(1-6) have higher polymerization and a greater number of branches.

Low-weight β-glucans are usually inactive, while those of intermediate weight have biological actions and their efficacy is low at cellular levels [8]. On the other hand, the molecular weight of β-glucans can directly activate leukocytes and stimulate their phagocytic, cytotoxic, and antimicrobial activity.

### Immunomodulatory and anticancer functions of β-glucans

Interestingly, the immunomodulatory and anticancer functions of β-glucans are also influenced by the conformation of the sugar chain, chemical modification, molecular weight, and kind of glycosidic bond. For instance, β-(1-3)-glucans have greater immunostimulatory effects than β-(1-6)-glucans and β-1,4-glucans [25,26]. The β-(1-3)-glucan, which was derived from *Saccharomyces cerevisiae*, improved the mice’s immune activity by decreasing the ratio of CD4^+^/CD8^+^ T cells and reduced the tumor progression in S180 tumor-bearing mice. Furthermore, β-(1-3)-glucan has increased the Bax expression and decreased the Bcl-2 expression, which leads to apoptosis in S180 tumor-bearing mice. Three types of soluble β-glucans (CG, SG, AG) are extracted from *Lentinus edodes*. They have a similar main chain of β-(1-3)-glucan and β-(1-6) branched structure. They can prevent the development of S-180 tumors in mice. AG has also been suggested to alter the tumor microenvironment by increasing the infiltration of CD4^+^ T cells or directly recruiting the neutrophils from peripheral blood to destroy tumors [27,28].

### Molecular weight efficacy

Molecular weight is another significant factor, influencing the physicochemical characteristics and biological activities of β-glucan. As a pathogen-associated molecular pattern (PAMP), β-glucan can modulate innate immune cells by binding to pattern recognition receptors (PRRs) on their surfaces. Seaweed's low molecular weight soluble β-glucan (Laminarin, 5300 Da) can exhibit an anticancer effect via mature dendritic cells [13,29]. A low molecular weight soluble β-glucan from yeast can also bind to the Dectin-1 receptor and exert an immunomodulatory effect. On the other hand, the high molecular weight β-glucan of oat indicates high viscosity, which diminishes its effects while the low molecular weight β-glucan from oat has been shown to substantially reduce cancer cell viability without any toxicity effect on normal cells [18]. Moreover, Artur javmen et al. isolated particle β-glucan from baker’s yeast *S. cerevisiae* and hydrolyzed it. They have found that soluble β-glucan of low molecular weight enhanced IFN-γ production more efficiently than particle β-glucan of high molecular weight [30,31].

Despite these, it is not yet clear whether molecular weight impacts β-glucan's antitumor immunity, as it is commonly assumed that the higher the molecular weight of β-glucan, the greater its anticancer effects would be. For instance, high-molecular-weight particle β-glucan generated by medicinal mushrooms has greater anticancer properties than low-molecular-weight soluble β-glucan.

### Branching degree and chemical modification

The activity of β-glucan is also affected by branching degree and chemical modification. The sulfated glucan (SGA) isolated from *Antrodia cinnamea* is a sulfated β-(1,4)-glucan. Each repeating unit has two long β-(1-6) branches. SGA was discovered to increase the cytotoxicity of cisplatin on lung cancer cells, decrease lung cancer cell proliferation and metastasis, and lower the tumor burden in lung cancer mice by blocking the TGF/FAK/PKB signaling pathway [32]. So, to summarize, β-glucan has a variety of sources and different structures, and its structure-activity relationship is complicated, and cannot be assessed using a single index.

## Potential receptors of β-glucan to fight against cancer cells

Due to the large molecular size of β-glucans, they cannot pass directly through the cell membrane. β-glucans as pathogen-associated molecular patterns (PAMPs) could be detected via cell surface receptors on innate immune cells such as macrophages and DCs [33]. The major receptors for β-glucans are Dectin-1 and the toll-like receptor (TLR). Other receptors include complement receptor 3 (CR3), scavenge receptors (SR), and lactosylceramide (LacCer) which are illustrated below [33,34].

## Dectin-1 receptor

Dectin-1 receptor is a lectin with 244 amino acids including four units of the extracellular domain, stalk, transmembrane region, and an intracellular cytoplasmic tail. The cytoplasmic tail contains an immunoreceptor tyrosine-based activation (ITAM)-like motif that activates tyrosine kinases. The dectin-1 receptor is mainly expressed in myeloid cells, such as neutrophils, DCs, and macrophages. It could detect the β-(1-3)(1-6) glucans from bacteria, fungi, and plants. The binding of β-glucans to dectin-1 activates several signaling pathways such as PI3K/Akt, MAPK, NFAT, and NF-κB that result in ROS production, phagocytosis, and cytokine secretion [35,36].

Several studies (in human and mouse models) have shown that higher stimulation of the Dectin-1 receptor is accomplished by particulate β-glucans compared to soluble β-glucans. Thus, particulate β-glucan increases cytokine secretion, respiratory burst (ROS production), and phagocytosis. This difference can be explained by the ability of particulate β-glucan to induce the clustering of the Dectin-1 receptors and exclusion of negative regulators of immune responses like CD148 and CD45 [37,38]. However, it does not mean that soluble β-glucan is unable to bind to the Dectin-1 receptor. Several studies have shown that low-molecular-weight soluble β-glucans (derived from yeast) can stimulate macrophages by binding to Dectin-1 thereby increasing the production of cytokines and ROS [39]. Moreover, β-glucans can activate DCs via Dectin-1 pathways, leading to inducing the differentiation of cytotoxic T lymphocyte and Th1 cells (cellular immunity) [20].

## Toll-like receptor (TLR)

TLRs are type I transmembrane receptors that are presented on macrophages, DCs, B cells, T cells, and endothelial cells. TLRs can recognize different microbes including fungi, bacteria, viruses, and protozoa through dsRNA, LPS, TLR5-Flagellin, etc. Interaction of ligands and TLRs induces several signaling pathways, such as MyD88- and TRIF-mediated signaling that triggers NF-κB and MAPK signaling as inflammatory signaling responses [40–42].

TLR4 could contribute to the enhanced activity of immune cells after exposure to β-glucans. According to the study by Sahasrabudhe et al., the level of NF-κB was increased following the stimulation with β-glucans in Dectin-TLR4 cell lines. A variety of β-glucans can bind to TLRs, such as zymosan binds to TLR2/4 of macrophages and by stimulating NF-κB signaling induces the production of cytokines like TNF-α and IL-12 [43]. Downstream signaling pathways of TLRs or dectin-1 may communicate with each other. Additionally, Zymosan by binding to both dectin-1 and TLR2 can trigger both dectin-1/Syk and TLR/MyD88 signaling pathways to induce the NF-κB subunits' translocation to the nucleus [44]. Isolated β-glucans from plants, including Phellinus linteus, Sparassis crispa, Platycodon grandiflorum, Angelica gigas Nakai, and Cordyceps millitaris, by binding to TLR4 induce maturation of DCs [45]. The binding of β-glucan to TLR4 has regulatory functions. An increased level of pro-inflammatory cytokines (IL-23, IL-4, IL-6, and TNF-α) was recorded in blocked TKR-4 receptors, which suggests the β-glucan as an immune regulatory ligand for TLR4 [28,38].

## Complement receptor 3 (CR3)

Complement receptor 3 (CR3) is a transmembrane heterodimer integrin consisting of two chains: CD11b (α_m_) and CD18 (β_2_). CD11b has a binding site for β-glucan. CR3 is mainly expressed in NK cells, monocytes, and neutrophils. Soluble and small molecular size β-glucan can bind to phagocytes (such as monocytes and neutrophils) and NK cells via CR3. This binding improves the phagocytosis and killing of tumors, which are opsonized through inactivated complement component 3b (iC3b) [35,46,47]. In other words, the binding of β-glucan to the C-terminal lectin domain increases adhesion to microbial cells and induces iC3b pathways resulting in tumor cytotoxicity and subsequently a cascade of cellular responses, including adhesion, phagocytosis, cytotoxicity, and migration [35,47]. Large molecular size β-glucan binds to TLRs, dectin-1 of macrophages, and DCs [45].

## Lactosylceramide (LacCer)

Lactosylceramide (LacCer) is expressed on neutrophils and endothelial cells. It has been identified as a receptor for β-glucan that recognizes several microbes and pathogens, including fungi. The binding of β-glucan with the LacCer receptor triggers various responses in neutrophils like chemotaxis, NF-κB activity, and cytokine secretion. These processes lead to the increased antimicrobial activity of neutrophils and induce the production of macrophage inflammatory protein-2 (MIP-2) and TNF-α via NF-κB and PKC signaling [48].

## Scavenge receptors (SR)

Scavenge receptors (SR) are present in epithelial, endothelial, and myeloid cells. Based on structure, SR is classified into A, B, and C classes. SR-A is a homo-trimeric transmembrane protein with an intracellular N-terminus, a cytoplasmic tail, a transmembrane sequence, and a spacer sequence. SR recognizes several ligands such as LDL, HDL, polyanionic molecules, as well as β-glucan [49]. Lentinan binds to the SR and induces signaling pathways including Akt kinase, PI3K, and MAPK [45].

**Figure 7:**
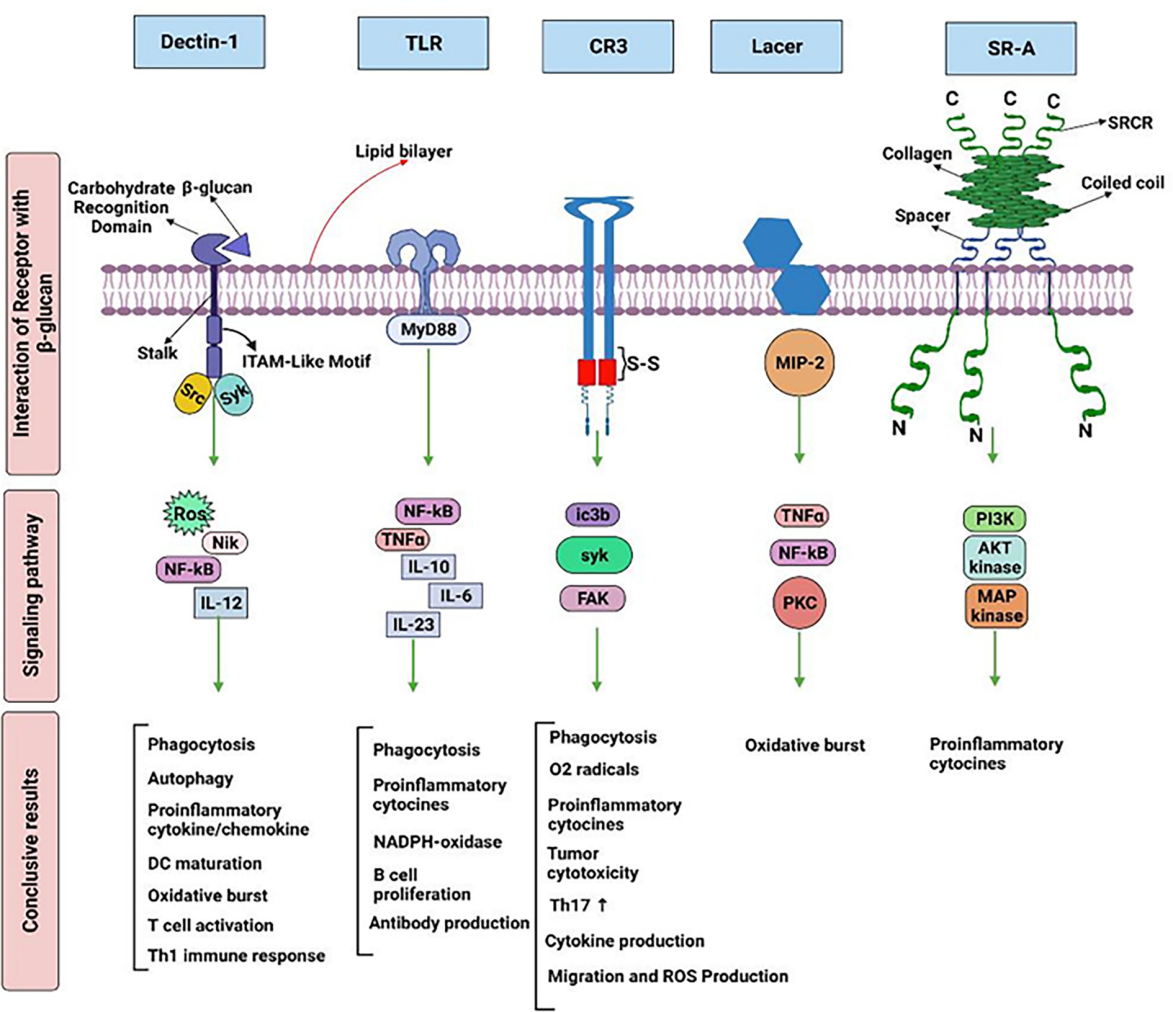
A demonstration of β-glucan cell surface receptors The structures of Dectin-1 and Toll-like receptor 2 (TLR2), the complement receptor 3 (CR3), Lactosylceramide (LacCer), and scavenger receptortype A (SR-A) are shown in interaction with β-glucan and subsequent downstream signaling pathways. ROS: reactive oxygen species; SRCR: scavenger receptor cysteine-rich.

Although the binding of β-glucan to LacCer or SR on the leukocyte cell surface has already been described, the biological mechanisms behind these interactions and their effects on the immune responses remain obscure and more investigations are required [28,50]. An overview of the mentioned β-glucan cell surface receptors is shown in Figure 7.

## Antitumor activity of β-glucan and probable mechanisms

Numerous *in vivo* investigations and animal-based *in vivo* studies have studied directly and indirectly antitumor effects of β-glucans, especially those extracted from *Lentinula edodes* (lentinan), *Grifola frondosa* (groan), *Sclerotinia scleratiorum* (Scleroglucan), and Schizophyllum commune (Schizophyllan) fungi [20]. Fungal β-glucans have emerged as a focus of research due to their potential anticancer properties. These complex polysaccharides, found in the cell walls of fungi, particularly medicinal mushrooms, have shown promise in fighting cancer. Several key points contribute to the discussion of their anticancer properties. First, fungal β-glucans have immunomodulatory effects. They can stimulate immune cells such as macrophages, NK cells, and dendritic cells, leading to enhanced immune responses against cancer cells. By activating the immune system, β-glucans improve tumor surveillance and aid in the elimination of cancerous cells. Additionally, β-glucans specifically activate and enhance the function of NK cells. These immune cells are capable of directly recognizing and eliminating cancer cells without prior sensitization. Activating NK cells through β-glucans can bolster their cytotoxic activity against cancer cells, thereby inhibiting tumor growth. Fungal β-glucans also interact with immune cell receptors, triggering immune signaling pathways. This interaction leads to the production of important cytokines, such as tumor necrosis factor-alpha (TNF-α), interleukin-6 (IL-6), and interferon-gamma (IFN-γ) [51]. These cytokines play pivotal roles in immune regulation and anticancer responses, further contributing to the potential anticancer effects of β-glucans. Moreover, β-glucans possess anti-inflammatory properties. Chronic inflammation is often associated with an increased risk of cancer development and progression. In addition to their immunomodulatory effects, fungal β-glucans may exert direct antitumor effects. Studies have indicated that they can inhibit cancer cell growth, induce apoptosis (programmed cell death) in cancer cells, and impede the formation of new blood vessels required for tumor growth (angiogenesis). Furthermore, β-glucans have shown potential in enhancing the efficacy of conventional cancer treatments, such as chemotherapy and radiotherapy. They may act synergistically by improving cancer cell sensitivity to these treatments or by augmenting the immune response against cancer cells [52,53]. Table 1 lists several β-glucans with their antitumor effects studied before.

**Table 1:** Antitumor and anticancer effects of β-glucans studies in previous studies

β-Glucan	Aim	System used	Results	References
β-glucan derived from fungal	This compound is isolated from *Omphalia lapidescens* and has antitumor activity	Sarcoma 180 in ICR mice	Anti-sarcoma tumor activity	[54]
Fungal β-glucan (OL-2)	Investigate the compositional properties of fungal (1→3)-β-D-glucan, OL-2, that isolated from *Omphalia lapidescens*	Sarcoma 180	Low anti-sarcoma (solid form) tumor activity, high anti-sarcoma (ascites form)	[55]
β-Glucan derived fungal (H-3-B; S-H-3-B)	Investigate the compositional properties of (1→3)-β-D-glucan, that isolated from *Cryptoporus volvatus*	Sarcoma 180 tumor	Anti-sarcoma tumor activity	[56]
Fungal β-glucan	Antitumor effect of a peptide-glucan extracted from *Agaricus blazei* in a double-grafted tumor system in mice	BALB/c mice	Notable activities related to chemotactic factor activity. Increase the IAP amount in the serum. Inhibited growth of Meth-A tumor	[57]
1–3, 1–4 oat β-glucan	oats in cancer cells-derived low-weight molecules that have anticancer activity, including Me45	Cancer cell lines	Induction of strong expression of caspase-12 in cancer cell lines (Me45, A431 and normal HaCaT and murine macrophages P388/D1) by β-glucan; significantly lower ABC reaction in HaCaT cells; negative ABC reaction in P388/D1 cell line	[18]
Fungal β-glucan	The therapeutic efficacy of β-glucans in patients who have cancer also as an adjunctive therapy in patients who received chemotherapy	Twenty patients with advanced malignancies	High tolerance of β-glucan in cancer patients receiving chemotherapy, which could have a positive effect on hematopoiesis	[58]
Commercial Sonifilan	Anticancer 1,3-β-glucan that cultured fruit body of *Sparassis crispa*	Sarcoma 180 in ICR mice	Anti-sarcoma (solid form) activity in ICR mice with a heavy vascular dilation and hemorrhage reaction	[59]
Grifolan LE (GRN), commercial Sonifilan	Antitumor effect of β-glucan that the cultured fruit body of *Agaricus blazei*	Sarcoma 180 in ICR mice	Anti-sarcoma (solid form) activity	[60]
Yeast β-glucan (WGP), soluble β-glucan (NSG), and barley β-glucan	Investigation of oral absorption of soluble barley and particulate yeast-1,3-glucan and its application to boost antitumor monoclonal antibody tumoricidal function in murine tumor models	C57BL/6 mice RMA-S-MUC1, a C57BL/6 lymphoma cell line	Similar function of orally administered yeast β-1,3;1,6-glucan to barley β-1,3;1,4-glucan with antitumor monoclonal antibody	[61]
Wellmune+antitumor mAb therapy	In cancer treatment, the therapeutic ability of different -glucan sources in combination with antitumor mAb	MyD88-deficient macrophages and DCs	When compared with mAb alone or mushroom-glucan extracts, the combination of yeast-glucan and antitumor mAb resulted in significantly lower tumor burdens and improved long-term survival	[62]
Lentinan	EROD activity, CYP1As levels, and NF-B and AhR DNA-binding activities were all measured.	BALB/c	The development of tumor necrosis factor-; an increase in the DNA-binding activity of nuclear factor-B suppresses the expression of lentinan hepatic CYP1As at both constitutive and inducible levels	[63]
Lentinan (L-FV-IB)	Correlation between antitumor activity, molecular weight, and conformation of lentinan	Sarcoma 180 (S-180) tumor cells	Maximum inhibition ratio against Sarcoma-180 solid tumor	[64]
Phycarine and lentinan	Immune reactions and the effects of marine 1,3 glucan	10-week-old BALB/c mice	Remarkable stimulation of phagocytic development, as well as potentiation of IL-1, IL-6, and TNF- production and release	[12]
Yeast p-β-glucan (WGP and PGG)	Pure soluble and particulate glucans derived from the yeast *S. cerevisiae*, as well as their modulatory effect on innate and adaptive immune responses, were determined	BMDC	The ability of particulate β-glucan to stimulate innate and adaptive immune responses through a dectin-1 pathway while soluble β-glucan primes innate neutrophils for tumoricidal activity via complement and CR3-dependent pathways.	[20]
β-Glucan from the Maitake aitake mushroom	β-Glucan, a polysaccharide contained in the Maitake itake mushroom, has been shown to have antitumor properties in prostatic cancer cells *in vitro*	Human prostate cancer PC-3 cells	The cytotoxic effect of a bioactive β-glucan from the Maitake mushroom	[65]
Mutated yeast β-glucan	Purified β-glucan (IS-2) from mutated *S. cerevisiae* has antitumor and immunostimulatory properties	Colon 26-M3.1 carcinoma and B16-BL6 melanoma cells	The activation of macrophages and natural killer cells inhibits lung tumor metastasis in a dose-dependent manner	[19]
Oat (Avena sativa) β-glucan	Evaluation of anticancer properties of radiation-degraded oat (*Avena sativa*) β-glucan	Colo-205, MCF7 and T47D cell line	The results showed that the highest cytotoxicity of γ-irradiated oat β-D-glucan was in cancer cell lines against COLO-205 and MCF7 cancer cells; No cytotoxicity against normal cell lines was observed at all concentrations	[66]
β-Glucan extracted from *Agaricus blazei* Murill	Investigation of anticancer properties of β-(1–6)-D-glucan which was achieved from *A. blazei* in an animal model, both *in vitro* and *in vivo*	A murine Lewis lung carcinoma cell line, 3LL	β-Glucan has cytotoxic properties for human ovarian cancer HRA cells but does not have this property for murine Lewis lung cancer 3LL cells, *in vitro*; β-glucan enhances p38 MAPK activity, which suppresses HRA cell proliferation and enhances the apoptotic pathway	[67]
β-Glucan from *Schizophyllum commune*	Evaluation of anticancer and macrophage properties of feed additives on β-glucan from *Schizophyllum commune* in breast cancer cells	Raw 264.7 cell line	Compared with the control group, the production of TNF-α and NO increased significantly after treatment with β-glucan for 24 h (*P*<0.05), indicating activation of macrophages; Also, induction of apoptosis by β-glucan inhibits breast cancer cell growth	[68]
Fungal β-glucan	Evaluation of the *in vivo* effects of β-glucan on peripheral blood monocytes and their expression of activation markers during short-term oral use in advanced breast cancer patients	23 female patients with advanced breast cancer	Peripheral blood monocytes-related proliferation and activity increased in patients with advanced breast cancer	[69]
*Candida albicans* β-glucan	*In vitro* evaluation of anticancer properties of *Candida albicans* β-glucan on MSCs supernatant to evaluate the apoptotic properties of lung cancer cells	MSC and lung cancer line	A significant decrease in MSC viability during 48 h in a dose-dependent manner (*P*<0.05); increased cancer cell apoptosis by the supernatant of mesenchymal stem cells treated with β-glucans	[70]
Fungal β-glucan	Investigation of anti-cancer features of β-glucan, vitamin C and Resveratrol.	BALB/c mice	The strongest activator of antibody formation and phagocytosis by combination, but not individual, components; The combination treatment strongly suppressed the growth of lung and breast tumors, most likely due to the stimulation of apoptosis	[71]
*Haesongi mushroom*	Investigation of nutritional constituents and anticancer features of *Haesongi mushroom* (*Hypsizigus marmoreus*)	Human cell line	The growth inhibitory properties of the water extract (5 mg/ml) on HepG2, AGS and SW480 human cancer cells were greater than ethanol extract.	[72]
Candida cell wall β-glucan	Evaluation of anti-cancer properties of *Candida* cell wall β-glucan on Lewis lung carcinoma cell line (LL/2) cells	Lewis lung carcinoma cell line (LL/2) cells	The results showed that β-glucan had a significant cytotoxic effect on both parental and sphere cell populations; Also, the expression of *Sox2* and *Oct4* genes decreased in β-glucan treated cells, which this decrease in gene expression was not observed in the control group	[73]
Fungal β-glucan	Investigation of the effect of β-glucan and adjuvant FOLFOX-4 on leukopenia and mucositis in 62 patients with colorectal cancer	62 consecutive patients with colorectal cancer treated with an adjuvant FOLFOX-4 regimen	Diarrhea and oral mucositis were less common in the β-glucan group; Also, the results showed that the harmful effects of chemotherapy were reduced by β-glucan	[74]
β-Glucan extract from *S.Cerevieses*	Anticancer effects of the β-glucan extract from the *S. crevices* on cancer cell lines	Two cancer cell lines (murine mammary adenocarcinoma AMN-3 cell line) and rat embryonic fibroblast (Ref) as normal cell line	The sulfation and carboxymethylation significantly enhanced the antitumor activities of the β-glucan against cell lines (AMN3, Sarcoma 180, and gastric carcinoma tumor cell *in vivo* and *in vitro*)	[75]
Fungal β-glucan	Evaluation of the effect of β-glucan on the life quality of women with breast cancer who have undergone chemotherapy	30 women with breast carcinoma	The results of β-glucan in breast cancer patients undergoing chemotherapy were improved Global health/QoL status and symptoms scales/ items, However, the change in Global health scores was not significant compared with the placebo group	[76]

## Direct anticancer effects of β-glucans

β-Glucans have a wide range of cytotoxic effects on cancer cells. They have a direct antitumor activity by blocking cell growth, inducing the cessation of the cell cycle in the phases and checkpoints of the cell cycle, increasing apoptosis, and controlling the signal transduction pathways associated with changes in major enzyme expression contributing to tumor progression. Growth inhibition by beta-glucans is reported in different cancer cell lines [77,78]. Various signaling molecules such as tyrosine-protein kinase (TPK), extracellular signal-regulated enzyme (ERK), cyclic adenosine monophosphate (cAMP), phosphatidylinositol (PI), nitric oxide (NO), and nitric oxide synthase (NOS) play significant roles in the induction of apoptosis in cancerous cells. Some β-glucans significantly induce the cell cycle arrest in the G1-phase due to the restriction of ERK1/2 or the ERK5 pathway, while others induce a gradual dose-dependent accumulation of cells at the G2/M phase along with a decrease in the population of cells in G1 phase [16].

Certain β-glucans have been shown to induce cell cycle arrest at the G1 phase. One mechanism through which β-glucans can achieve this effect is by restricting the activity of the ERK1/2 pathway. The ERK1/2 pathway is part of the mitogen-activated protein kinase (MAPK) signaling cascade, which regulates cell proliferation, survival, and differentiation. Inhibition of the ERK1/2 pathway by β-glucans can disrupt the signaling events that drive G1 phase progression, leading to cell cycle arrest [79,80]. When the ERK1/2 pathway is inhibited, it can interfere with the activation of downstream transcription factors involved in cell cycle progression, such as members of the E2F family. These transcription factors regulate the expression of genes required for DNA synthesis and cell cycle entry. By restricting ERK1/2 activity, β-glucans can impede the transcriptional activation of these genes, thereby preventing cells from transitioning from the G1 to S phase, where DNA replication occurs [81]. In contrast to G1 phase arrest, some β-glucans have been found to induce a gradual accumulation of cells at the G2/M phase of the cell cycle. This effect is typically observed with higher doses of β-glucans. The mechanisms underlying G2/M phase arrest induced by β-glucans are not as well understood compared with G1 phase arrest, but several possibilities have been proposed [82,83].

In cancerous cells, β-glucans can also induce apoptosis by inhibiting the telomerase activity, affecting the mitochondrial membranes, and altering the expression and activity of apoptosis-related genes, such as caspase 3 and 9 p53 [84,85]. Pro-apoptotic properties of (1,3;1,4)- Oat β-D-glucan from *Avena sativa* have been reported against human melanoma HTB-140 cells. It increases the caspase-3/7 activation and prevalence of phosphatidylserine on the external membranes, which indicates induction of the mitochondrial pathway of apoptosis through ugh reduction in intracellular ATP levels [86]. Choromanska et al have also reported a new and low-MW potent anti-cancer (1,3;1,4)-β- β-glucan from oats. The glucan- may induce strong expression of caspase-12 in Me45 and A431 cancer cell lines causing apoptosis [18]. On the other hand, the normal physiological function of cells is highly dependent on the biochemical properties s of the cell membrane. Any change in normal cells may cause oncogene activation and induction of tumors. On the other hand, alterations in the content of tumor cells could result in cell death. Polysaccharides are reported to affect the cell surface receptor rs, material transport, and block the proliferation and angiogenesis of tumor cells by increasing the sialic acid content of the tumor cell membrane and down-regulation of VEGF and VEGFR expression in tumor vascular cells, respectively [84].

## Indirect anticancer and immunomodulatory effects of β-glucan

β-Glucan-mediated immune modulation depends on (i) intestinal absorption, (ii) body circulation, and (iii) immune cell activation at distant lymphoid organs [87]. In comparison with the peptide-, protein-, virus-, and virus-like-particle-based immune modulators, β-glucan provides numerous advantages including (i) β-glucans are non-immunogenic molecules because their lack of peptide and protein content makes them unlikely to trigger non-specific immune responses; (ii) β-glucans are non-toxic. Even high doses of up to 10 mg/kg are well tolerated *in vivo* with no adverse effects; (iii) the immunomodulatory properties of β-glucan are specific as it mediates its stimulatory activity on immune cells using specific receptors on the surface of them as we discussed before; (iv) β-glucans have several aldehydes and hydroxyl groups, providing opportunities to modify structures, improve physiological characteristics, and synthesis of β-glucan-based nanoparticle system with potential for carrying a high level of agents with immune-modulatory effects [88].

Polysaccharides like β-glucans can activate different types of immune cells (T-cells, B-cells, MT, NK cells, etc.), induce the removal of pathogens and aged cells, and trigger the production of various cytokines and interleukins [89]. Furthermore, investigations suggest that β-glucans are potential immune modulators by manipulating TME innate and adaptive immune cell response and enhancing clinical outcomes for immunotherapies for cancer [88].

Macrophages could be activated by polysaccharides. These cells could enhance the phagocytosis by lymphocytes, secretion of NO, production of TNF-α, and many other cytokines, which could lead to the increased anti-tumor activity of immune cells. β-glucans mediate the activation of lymphocytes (T, B) as another immunomodulatory function. Activation of T cells increases the phagocytosis of tumor cells via other immune cells such as macrophages and natural killer cells. β-Glucans also induce the proliferation of peripheral blood mononuclear cells (PBMC) and stimulate macrophages and dendritic cells (DCs) to produce cytokines and chemokines, including TNF-α, IL-12, CXCL2, IL-6, IL-1β, and IL-10. They also could induce the cytotoxicity of NK cells against various cancer cell lines by activation of NKG2D/NCR receptors and MAPK signaling pathways. These processes eventually lead to exocytosis of perforin and granulysin [90,91]. Lentinan could increase cytotoxic activity and inflammatory cytokines of primary macrophages, and maturation of dendritic cells (DCs) via IL-12 production [92]. Lentinan could act as a vaccine adjuvant that enhances T-cell functions in tumor-modeled mice and cancer patients. Of note, macrophages and DCs are activated by β-glucan directly, while lentinan activated T-cells indirectly via IL-12 and IFN-γ produced by macrophages and DCs [45,93]. It has been also reported that fungal glucans can activate neutrophils and enhance efficient phagocytosis [6]. For instance, a study has shown that pre-treatment of mice with β-glucan reduced tumor progression. In this regard, the anticancer impact of β-glucan-induced immunity was coupled with epigenetic and transcriptome rewriting of neutrophils and granulopoiesis reprogramming toward an anticancer phenotype. This mechanism needed type I interferon signaling regardless of host adaptive immunity. β-Glucans are also reported to promote antibody production and complement activation, which could lead to the activation of NK, T, and B lymphocytes, immunopotentiation, and inhibition of VEGF by promoting the complement production [94]. Up-regulation of TLR2 in DCs is the other reported immunomodulatory effect of β-glucans. This up-regulation could promote the expression of CD40, CD80, CD86, and MHC Class II (protein-bound polysaccharide activates DCs and enhances OVA-specific T-cell response as a vaccine adjuvant) and improve the fluidity of erythrocyte membrane [95].

## Modulation of myeloid immune cells: macrophages, monocytes, DCs, and neutrophils

Phagocytes (such as monocytes/macrophages, DCs, and neutrophils) are the most important myeloid cells in the innate immune system. Monocytes and macrophages are capable of inflammatory cytokines secretion, antigen presentation, and phagocytosis. These cells play a linking role between innate immunity and adaptive immunity. Macrophages of innate immunity can phagocytize self-mutated cells and pathogens. In this regard, macrophages can experience M1/2 type activation in various environments, which is linked to tumor incidence, progression, treatment resistance, and metastasis. M1 macrophages can release pro-inflammatory molecules and have anti-cancer effects, while M2 macrophages improve tumor development and metastasis [96]. They also can phagocytize most thymus-dependent (TD) antigens and process them as antigen-presenting cells (APCs).

Many adhesion molecules (on the surface of macrophages) can engage with receptors of co-stimulatory molecules (on the surface of T cells) to create co-stimulatory signals, activate T cells, and begin adaptive responses.

DCs and macrophages are the basic targets of β-glucans. The repetitive structures of these polysaccharides can bind to the surface receptors of various immune cells, such as natural killer (NK) cells, neutrophils, macrophages, and B- and T- T-lymphocytes. The interaction between these molecules and the corresponding receptors activates the immune system against pathogens and cancer cells [97,98]. Once β-glucan is recognized by immune cell receptors, it stimulates innate and adaptive immunity (by activating different signaling pathways) and leads to an increased rate of antigen presentation, phagocytosis, chemokine/cytokine secretion, and production of reactive oxygen species (ROS) [99]. Imprime PGG (a kind of soluble β-(1-3) (1-6)-glucan) can bind to CD11b on myeloid cells (monocytes or neutrophils), which increases the anti-tumor immune response stimulation in M1 macrophages and N1 neutrophils. They also decrease the immunosuppressive function of tumor-associated macrophages (TAM), M2 macrophages, N2 neutrophils, and bone marrow-derived suppressor cells (MDSCs). Moreover, Imprime PGG can boost the anti-cancer activity of immunological checkpoint antibodies, anti-angiogenic antibodies, and tumor-specific antibodies by inducing chemokines, stimulating innate immune effector cells, and collaborating with adaptive immune cells [91,100]. TAM is crucial in the progression of malignant solid tumors, including breast cancer [101]. Polysaccharide-based drug delivery systems involving mushroom, yeast, and hyperbranched β-d-glucans have been explored for their applications as nano-carriers for targeting TAM. TAM is more complex than M2-like macrophages, and their signaling pathways are triggered by factors within TAM. Mushroom polysaccharides can enhance immune suppression in a Lewis lung carcinoma model, while zymosan can reshape TAM into producers of inflammatory chemoattractants. Transcription factor EB (TFEB) overexpression can modulate TAM gene expression and function through multiple pathways, making it a promising approach for improving the efficacy of existing treatments, including immunotherapies for breast cancer [101–103].

It has been revealed that the anticancer activity of β-glucan is also linked to the activation of the complement system. Chan et al. have found that Imprime PGG binds to endogenous β-glucan antibodies in plasma after intravenous administration. This interaction activates the complement and leads to the deposition of complement protein (iC3b) in the tumor to create a tripartite immunological complex (Imprime PGG/ABA/iC3b).

These complexes connect to the innate immune effector cells via complement receptor 3 (CR3) and the Fc portion of immunoglobulin G receptor IIa (FcgRIIa) and improve the activities of immune effector cells (such as stimulation of antibody-dependent phagocytosis, which is mediated by macrophages and stimulation of ROS production by neutrophils) [104].

BG136, a β-(1-3)(1-6)-glucan derived from *Durvillaea Antarctica*, is also suggested to have antitumor activities. This property is due to its ability to boost the anticancer effect of anti-PD-1 antibodies, modulate the number of immune cells in the tumor microenvironment (TME), increase the macrophage phagocytosis, regulate the B-lymphocytes activity, and enhance the pro-inflammatory response of AW264.7 macrophages. BG136 can activate the RAW264.7 cells, which express low Dectin-1. Activation of these cells induces the TLR4-dependent expression of NF-κB and increases cytokine expression [105].

## Modulation of lymphoid immune cells: NK cells and T lymphocytes

Natural killer T (NKT) cells, cytokine-induced killer cells, and NK cells are some crucial innate effector cells in the immune surveillance of cancer. They function as a bridge between innate and adaptive immunity and have a stronger influence on anticancer immune responses. NK cells are a type of congenital lymphoid cells, which display several active receptors. They can identify soluble markers on the surface of cancer cells and kill them without sensitization. NK cells are important cytotoxic effector cells of innate immunity. They account for approximately 5–15% of all circulating lymphoid cells, which are activated at TME, stimulate the adaptive immunity (via direct cytotoxicity or release of cytokines such as IFN-γ), and participate in the eradication of tumor cells [106].

Paramylon is a linear β-D-glucan, which is derived from *Euglena gracilis* (EG). It stimulates the immune system by increasing the expression of CD69 and the production of proinflammatory cytokines (IL-1β, IL-6, TNF) by NKT and NK cells [107]. Prior findings indicated that β-glucan binds to NKp30 (a microbial pattern recognition receptor) and directly activates NK cells. β-(1-3)-glucan stimulates the production and clustering of NKp30 at the microbial and NK cell synapses. This clustering event promotes the perforin release for fungal cytotoxicity via Src Family Kinase signaling [108]. Furthermore, another key β-(1-3)-glucan receptor, CR3, is highly expressed on monocytes, neutrophils, and NK cells, while its expression is lower on macrophages [109].

Cytokines play a critical role in the immune system by mediating communication between cells and regulating various immune responses. They are small proteins or glycoproteins produced by immune cells and other cell types, acting as signaling molecules.

One important mechanism in which cytokines function is inflammation. Inflammatory cytokines, such as tumor necrosis factor-α (TNF-α) and interleukin-1 (IL-1), are produced in response to infection, tissue damage, or immune triggers [110,111]. They initiate and regulate inflammatory responses by promoting the recruitment of immune cells to the site of inflammation, enhancing vascular permeability, and activating immune cells to eliminate pathogens or damaged cells. These cytokines play a pivotal role in initiating and regulating inflammatory responses. For instance, when tissue damage or infection occurs, immune cells and damaged cells release inflammatory cytokines into the surrounding area. These cytokines act as signaling molecules, facilitating communication between cells involved in the immune response. They bind to specific receptors on immune cells, triggering a cascade of events that lead to inflammation. One of the primary functions of inflammatory cytokines is to promote the recruitment of immune cells to the site of inflammation. They act as chemical messengers, attracting immune cells such as neutrophils, monocytes, and lymphocytes towards the affected area. This recruitment is crucial for the immune cells to reach the site of infection or tissue damage and initiate the appropriate immune response. Inflammatory cytokines also enhance vascular permeability, which means that they increase the leakiness of blood vessels in the inflamed area. This increased permeability allows immune cells and other molecules to exit the blood vessels and enter the affected tissue more easily. This process facilitates the delivery of immune cells and mediators to the site of inflammation, enabling them to combat pathogens or repair damaged tissue [112,113].

Cytokines also play a crucial role in the activation and differentiation of immune cells. For example, interleukin-2 (IL-2) is a key cytokine that promotes the proliferation and activation of T cells, B cells, and natural killer (NK) cells. It supports the development and maintenance of regulatory T cells (Tregs), which help regulate immune responses [114]. For example, IL-2 is known for its ability to promote the proliferation and activation of various immune cell types, including T cells, B cells, and NK cells. When immune cells encounter specific antigens, IL-2 is produced and acts as a growth factor, stimulating the expansion and activation of these cells. T cells, a key component of adaptive immunity, are particularly influenced by IL-2. Upon activation, T cells express IL-2 receptors on their surface, allowing them to respond to IL-2 signaling. IL-2 acts as a potent T-cell growth factor, supporting their proliferation and differentiation into effector T cells, which carry out immune functions such as killing infected cells or coordinating immune responses. IL-2 also plays a role in the development and maintenance of Tregs. Tregs are a specialized subset of T cells that help regulate immune responses and maintain immune tolerance. IL-2 signaling is crucial for the survival and function of Tregs, as they rely on IL-2 for their development and maintenance. IL-2 promotes the differentiation of Tregs and supports their suppressive function, which helps prevent excessive immune responses and maintain immune homeostasis [115–117].

Furthermore, cytokines facilitate communication between different immune cells. They can act in an autocrine manner, where a cell secretes cytokines that bind to receptors on its own surface, resulting in self-stimulation. Cytokines can also act in a paracrine or endocrine manner, where they are secreted by one cell and bind to receptors on neighboring or distant cells, respectively, to induce specific responses [118]. Cytokines can modulate immune responses by promoting or suppressing specific immune functions. For instance, interferon-γ (IFN-γ) enhances the activity of macrophages, promotes the differentiation of T helper 1 (Th1) cells, and boosts antigen presentation. Conversely, transforming growth factor-β (TGF-β) is an immunosuppressive cytokine that inhibits immune cell activity and promotes immune tolerance [119,120].

**Figure 8:**
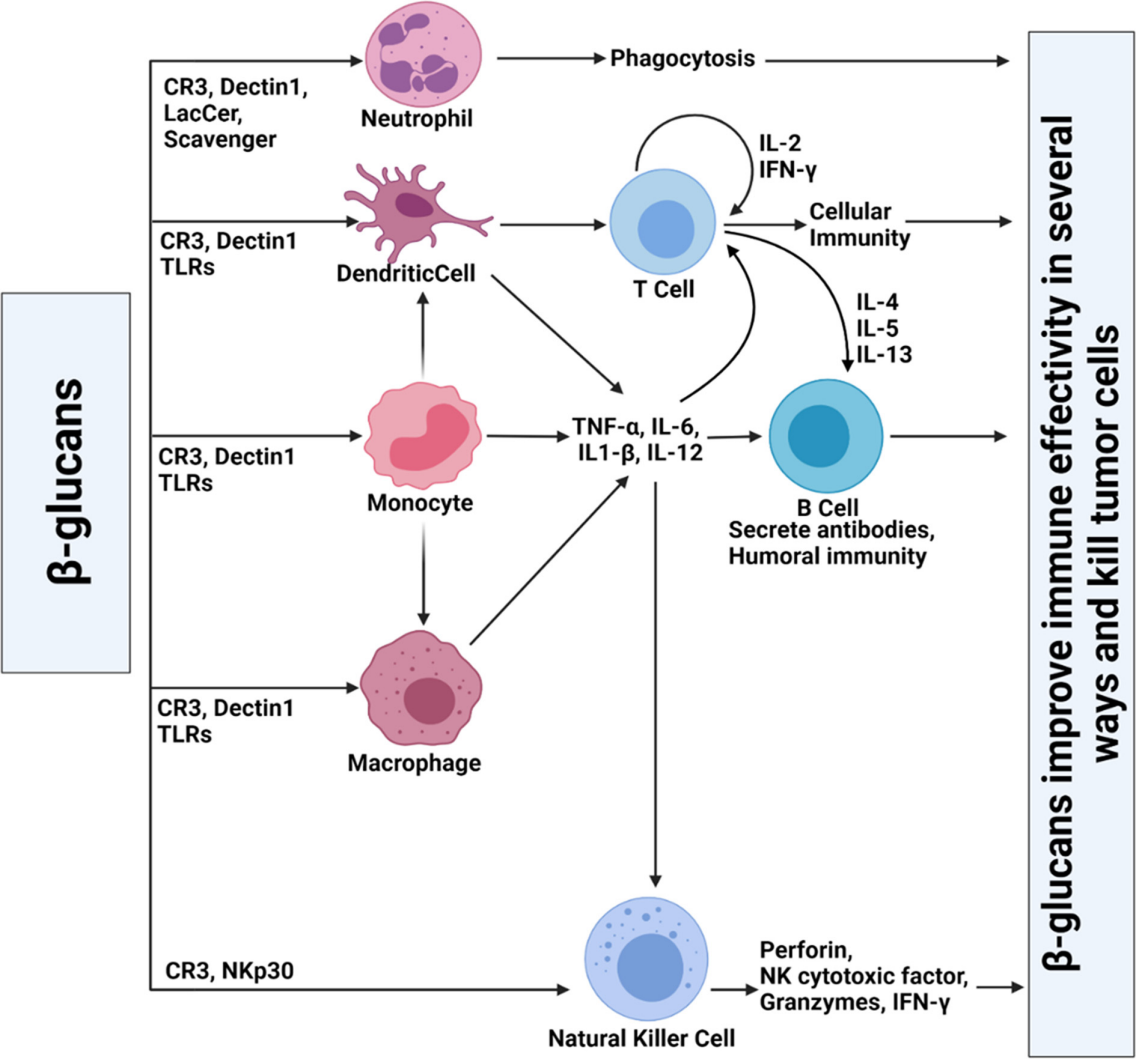
The antitumor mechanism of β-glucans β-Glucans can improve the immune effectivity in numerous pathways including stimulation of NK cells, production of inflammatory cytokines, and sequential activation of B and T cells (as shown in this diagram) that leads to tumor cells killing.

*Grifola frondosa* polysaccharide (GFP) is a soluble β-glucan, which has been demonstrated to increase the proliferation of lymphocytes and NK cells in immunosuppressive mice. GFP prevents bone marrow suppression and immunosuppression via increased expression of kinases and transcription factors (p-JAK2/JAK2, p-STAT3/STAT3, and SOCS3) in the JAK2/STAT3/SOCS signaling pathway. Therefore, GFP activates the macrophages and NK cells, efficiently increases T-cell proliferation, and augments T-cell activity in cellular immunity. Moreover, GFP can enhance the production of IFN-Ɣ and TNF-α to strengthen humoral immunity. Thus, GFP can modulate immune activity through improved proliferation of immune cells (macrophages, NK cells, CD8^+^ T cells, and CD4^+^ T cells) and secretion of immune factors (IFN-γ and TNF-α) [121,122]. Figure 8 illustrates the immunomodulatory activities of β-glucan.

## Conclusion and future perspective

Antitumor drugs often cause side effects. As a result, they have little impact on the treatment process. Of note, when these drugs are combined with β-glucan, their treatment effect is doubled, and there is no poisoning [123].

Evidence has revealed that these compounds kill cancer cells. Due to their antitumor function, fungal β-glucans have been used in cancer therapy of patients for nearly 50 years, and clinical trials in this field are still ongoing. β-glucans bind to receptors on macrophages and NK cells and stimulate the cells to attack cancer cells. When β-glucans enter the bloodstream, the cells release various factors such as TNF (tumor necrosis factor), IL-1 and IL-6, hydrogen peroxide (H_2_O_2_), and interferon into the bloodstream, which all fight cancer and other infections. Studies have uncovered that some fungal-glucans are beneficial to the development and progression of cancer and cause small tumor masses in experimental mice. Fungal β-glucans, together with monoclonal antibodies, have synergistic effects on treating cancer [124]. Treatment with monoclonal antibodies has been shown to target compounds involved in carcinogenesis. Moreover, a combination of these antibodies with fungal β-glucans has been suggested to enhance neuroblastoma tumor regression and prolong lifespan in experimental mice.

β-glucans, such as Wellmune Product Glucan (WPG), promote the regression of tumors by stimulating granulocytes and macrophages, which in turn poison the tumor cells. Increasing the patients’ resistance and endurance as well as speeding up the healing of complications due to chemotherapy’s toxic effects, especially leukopenia, which elevates the risk of infection, contribute to the treatment process. Moreover, the intraperitoneal injection of some yeast-derived β-glucans reduces the mortality of tumor-bearing mice exposed to the whole body and increases the number of leukocytes (white blood cells) and lymphocytes [125].

Cancer has been reported to be successful after surgery, leading to increased survival in mice, and lentinan similarly elevates the life expectancy of cancer patients by an average of 199 to 297 days.

Structure and activity are relevant in β-glucans. The available literature revealed that the Mw of a β-glucan has a major influence on its physicochemical properties and affects its biological activities [126]. Short β-glucans with a molecular weight of 5–10 kDa are generally inactive. Although unbranched β-glucan has good biological activity, the chemical addition of β-(1-6) glucose residues to the curdlan backbone increased the antitumor activity, as highly branched β-glucan has a higher affinity for receptors. Soluble β-glucans are stronger immunostimulators than insoluble ones [45].

Studies on the anticancer effects of β-glucans have disclosed that the polysaccharide β-glucans has an inhibitory influence on the growth of tumor cells in the body [127]. They can also affect the expression of several genes associated with cancer cells can stop the cell cycle of cancer cells and induce an apoptotic reaction in these cells [128]. It should be noted that hydroxymethylation, hydroxypropylation, and methylation of β-glucans can increase their water solubility and antitumor activity. Jabber et al., in their study on the anticancer effects of β-glucan extracted from *S. cerevisiae*, have pointed out the high-water solubility and great anticancer activity of the methylated carboxyl derivatives of both α- and β-glucan compounds [75].

Weitberg et al. investigated the therapeutic effect of fungi β-1,3- and β-1,6-glucan on cancer patients; also, adjuvant therapy in patients undergoing chemotherapy to suppress hematopoiesis. In their study, all the patients who had advanced malignancy and underwent chemotherapy received β-glucan, and their condition was monitored in terms of tolerance and effect on hematopoiesis. Their results demonstrated that cancer patients undergoing chemotherapy could tolerate fungal β-glucan, which is likely to suggest the beneficial effect of this polysaccharide on hematopoiesis in these patients [58]. The impact of yeast β-glucan on the quality of life was examined by Ostadrahimi et al. in a study on women with breast cancer undergoing chemotherapy. The patients received daily commercial yeast β-glucan capsules (10 mg) for 21 days. Based on the results, they concluded that β-glucans could act as a proper adjunct in chemotherapy and ameliorate such patients’ quality of life [76]. β-Glucans are known as biologically active natural fibers or polysaccharides whose importance in medicine and health, including antitumor and anticancer activity, has been proven over the years. These bioactive compounds are safe for oral use not only as a dietary supplement but also as a part of a daily diet. In this review, we discussed the antitumor and anticancer activity of β-glucans examined in previous surveys. The medical importance and effectiveness of β-glucans have been corroborated *in vitro* and through clinical trials on animals and humans. β-glucan significance has not yet been studied systematically, clinically, and physiologically which needs to be considered by future studies.

However, through the identification of downstream signaling pathways, and understanding the interaction of β-glucan with its respective receptors in greater detail, we can widely use β-glucans in the treatment of infectious diseases and cancers.

## Data Availability

The datasets used and/or analyzed during the current study are available from the corresponding author upon reasonable request.

## Abbreviations

BRM: biological response modifier
cAMP: cyclic adenosine monophosphate
CR3: Complement receptor 3
DC: dendritic cell
EG: Euglena Gracilis
Erk: extracellular signal-regulated enzyme
FcgRIIa: Fc portion of immunoglobulin G receptor IIa
GFP: Grifola frondosa polysaccharide
iC3b: inactivated complement component 3b
ITAM: immunoreceptor tyrosine-based activation
MDSC: marrow-derived suppressor cell
Mo-DC: monoclonal-dendritic cell
NKT: natural killer T cell
NO: nitric oxide
NOS: nitric oxide synthase
PAMP: pathogen-associated molecular pattern
PBMC: peripheral blood mononuclear cell
PI: phosphatidylinositol
PRR: pattern recognition receptor
ROS: reactive oxygen species
TLR: Toll-like receptor
TME: tumor microenvironment
TPK: tyrosine-protein kinase
WPG: Wellmune Product Glucan
